# The presence of ACE2 and regulatory miRNAs (miR-200c-3p and miR-421-5p) in the saliva of periodontitis patients post-COVID-19 vaccination

**DOI:** 10.3389/fdmed.2024.1438139

**Published:** 2024-09-04

**Authors:** Boy M. Bachtiar, Natalina Haerani, Yuniarti Soeroso, Nada Ismah, Endang W. Bachtiar

**Affiliations:** ^1^ Department of Oral Biology and Oral Science Research Center, Faculty of Dentistry, Universitas Indonesia, Jakarta, Indonesia; ^2^ Department of Periodontology, Faculty of Dentistry, Universitas Indonesia, Jakarta, Indonesia; ^3^ Department of Orthodontics, Faculty of Dentistry, Universitas Indonesia, Jakarta, Indonesia

**Keywords:** COVID-19 vaccine, periodontitis, miR-200c-3p, miR-421-5p, IL-6

## Abstract

The effectiveness of COVID-19 mRNA vaccines in individuals with periodontitis is crucial. This study evaluated the efficacy of the BNT162b2 vaccine in individuals with periodontitis who had been vaccinated at least 6 months earlier. Using real-time PCR, the association between the SARS-CoV-2 receptor angiotensin-converting enzyme 2 (ACE2) and miRNA-200c-3p and miRNA-421-5p as well as interleukin-6 (IL-6) was examined in the saliva of moderate (G1, *n* = 10) and severe (G2, *n* = 10) periodontitis subjects. Participants without periodontitis were included as a control group. The transcription levels of soluble ACE2 and IL-6 were higher in periodontitis participants than in control participants, but within periodontitis groups, only IL-6 expression was higher in G2 than in G1. A positive strong correlation between ACE2 and IL-6 was only observed in the G2 group (*p *= 0.008). The expression of miR-200c-3p but not miR-421-5p was higher in periodontitis individuals. Their relationship was positive but a strong correlation was only observed in the G2 group. In all periodontitis groups, a strong inverse correlation was observed between the two microRNAs and ACE2. However, receiver operating characteristic (ROC) analysis showed that only the relationship between ACE2 and miR-4215p had potential as a biomarker for the efficacy of the mRNA vaccine, with areas under the ROC curve of 0.92 and 0.80 in the G1 and G2 groups, respectively. Our study revealed that active and non-active periodontitis conditions do not interfere with the efficacy of the BNT162b2 vaccine for at least 6 months post-vaccination. This suggests that in individuals with periodontitis, soluble ACE2 in the saliva may serve as a preliminary indicator of vaccine response.

## 1 Introduction

In January 2020, the World Health Organization (WHO) declared the discovery of a novel coronavirus known as SARS-CoV-2 (1). SARS-CoV-2 is a virus responsible for COVID-19, a new infectious respiratory disease with an emergent outbreak that impacts all populations (2).

Although vaccination is a promising strategy for preventing SARS-CoV-2 infection, its efficacy is crucial, particularly for vulnerable people, such as those with periodontitis, an oral tissue inflammation that can be a modifiable factor for COVID-19 complications (3, 4). This is because the oral cavity is a reservoir for SARS-CoV-2 (5), as the virus can be found in the periodontal pocket (6), gingival crevicular fluid, and saliva (7, 8).

However, an earlier study demonstrated that 6 months post-COVID-19 vaccination, the IgA against the SARS-CoV-2 N protein can be detected in the oral fluids of vaccinated individuals (9). This indicates that the long-lasting mucosal immune response may reflect the effectiveness of COVID-19 vaccines in reducing or minimizing the risk of SARS-CoV-2 infection. As the vaccines are administered to all healthy people and the pathogenic action of the mRNA-based vaccine and SARS-CoV-2 is similar (10), it is reasonable to expect a difference in the biological effect due to the intramuscular administration of the vaccine, especially when considering that periodontitis may occur concurrently and be unrelated to the vaccine.

In addition to antibodies against SARS-CoV-2 (11), vaccine efficacy can be analyzed through the expression of microRNA (miRNA) (12); the expression levels of numerous miRNAs are altered after COVID-19 vaccination (13). Apart from their known functions inside cells, miRNAs can be reliably found in bodily fluids, such as saliva, where the majority of detectable miRNA is located in exosomes (14). Moreover, a previous report indicated that miRNA-200c-3p and miRNA-421-5p regulate the expression of angiotensin-converting enzyme 2 (ACE2), which is the primary cell entry receptor for SARS-CoV-2 during COVID-19 infection (15). This ACE2 receptor is abundantly expressed in a number of organs, including the salivary glands (16). Therefore, the aim of the present study was to assess the relationship between ACE2 and its regulators (miRNA-200c and miR-421) in individuals with periodontitis after receiving booster mRNA vaccines (BNT162b2). Interleukin-6 (IL-6) was selected for analysis because it interacts with ACE2 regulators (17, 18), contributes to the development of periodontal disease, and maintains periodontal tissue homeostasis by coordinating the host response (19).

## 2 Material and methods

### 2.1 Recruitment of study participants

This observational study was conducted from May to July in 2023 after participants provided their consent (ethics approval ref. 90/Ethical Approval/FKG UI/X/2022) before saliva collection. In addition, to perform this study, we followed the guidelines of the Strengthening the Reporting of Observational Studies in Epidemiology (STROBE) statement (20). Information regarding the clinical data was obtained from hospital reports (data not shown). All recruited participants in this study were individuals who had received the BNT162b2 vaccine (either the Pfizer-BioNTech COVID-19 or the Moderna COVID-19 vaccine) at least 6 months before the study started. Participants with no previous SARS-CoV-2 infection (aged > 18 years) were included in this study. Except for patients aged >60 years, no exclusion criteria were applied. All participants had received the first and second doses of the COVID-19 vaccine (Sinovac-CoronaVac vaccine), which was confirmed through the application (PeduliLindungi app) provided by the Indonesian government (21).

Of the 30 participants, 10 had moderate periodontitis (**G1**), 10 had severe periodontitis (**G2**), and 10 did not have periodontitis (controls). Moderate to severe periodontitis was diagnosed according to the established criteria (20).

### 2.2 Salivary sample collection

A recent report showed that salivary components can be used as surrogates for systemic biomarkers (22). In this study, unstimulated saliva (1 ml) was collected from all recruited participants. The saliva was collected between 8 a.m. and 11 a.m., during which time the subject had been requested to refrain from food and drinks (for at least 1 h). The saliva was collected using a sterile pipette and immediately put on ice and stored at −80°C until further use.

### 2.3 Quantification of ACE2 and IL-6 transcription by quantitative real-time PCR

RNA was extracted from saliva using GENEzol™ (General, Ltd, New Taipei City, Taiwan), as per the manufacture's recommendations, followed by reverse transcription using an Applied Biosystems kit (Waltham, MA, USA). The resulting cDNA was amplified by quantitative real-time PCR (qPCR) with specific primers; non-transcribed RNA was used as a control for genomic DNA contamination. The resulting cDNA was amplified in triplicate using an ABI StepOnePlus Real-Time PCR system with SYBR Green PCR Master Mix (Applied Biosystems). The PCR primers for ACE2 were as follows: forward, 5′-ACAGTCCACACTTGCCCAAAT-3′, and reverse, 5′-TGAGAGCACTGAAGACCCATT-3′ (23). For IL-6, the forward and reverse primers were 5′-GATTCAATGAGGAGACTTGCC-3′ and 5′-GGTCAGGGGTGGTTATTGC-3′, respectively (15).

The PCR conditions were as follows: pre-denaturation at 95°C for 5 min, followed by 40 cycles of 95°C for 10 s, 60°C for 30 s, and 72°C for 30 s, and a final extension at 72°C for 5 min. Gene expression was normalized to the level of glyceraldehyde-3-phosphate dehydrogenase (GAPDH); the forward and reverse primers were 5′-AATGGAAATCCCATCACCATCT-3′ and 5′-CAGACTCGCACTTG-3′, respectively (24). The mRNA expression of each target gene in the saliva from vaccinated participants without periodontitis was set as a control. Results greater or less than 1 were considered to indicate the upregulation or downregulation of mRNA expression. All values obtained from the tested participants with periodontitis were standardized and compared with the control value, represented as a value of 1.

### 2.4 Exosome

The exosomes from saliva specimens of patients with periodontitis and controls were extracted using EXOquick-TC^™^ (EQ, System Biosciences Inc., Mountain View, CA, USA), according to the manufacturer's instructions. Total RNA from exosomes was extracted using GENEzol™ reagent (General, Ltd, New Taipei City, Taiwan) as per the manufacturer's recommendations. The final concentration of purified RNA was determined using Qubit assay reagent (Invitrogen, Carlsbad, CA, USA) followed by reverse transcription using an Applied Biosystems kit. The resulting cDNA was amplified in triplicate using an ABI StepOnePlus Real-Time PCR system with SYBR Green PCR Master Mix (Applied Biosystems). The forward and reverse primers for miR-200c-3p were 5′-GCGGCGGTGGCAGTGTCTTAGC-3′ and 5′-ATCCAGTGCAGGGTCCGAGG-3′, respectively (15). The forward and reverse primers for miR-4215p were 5′-GCTTCGGCAGCACATATACTAAAAT-3′ and 5′-CGCTTCACGAATTTGCGTGTCAT-3′ (15), respectively. Using the forward (5′-CTCGCTTCGGCAGCACA-3′) and reverse (5′-AACGCTTCACGAATTTGCGT-3′) primers (25), the expression of each miRNA was normalized to U6 snRNA and standardized to the control value as mentioned above.

The PCR conditions were as follows: 95°C for 2 min, followed by 35 cycles of 95°C for 5 s, 62°C for 10 s, and 72°C for 30 s, then 95°C for 1 min, 62°C for 1 min, and 96°C for 15 s.

### 2.5 Statistical analysis

Analysis of the descriptive data was performed using GraphPad Prism software, version 10.1.0. (San Diego, CA, USA), and was presented as median (range). One-way ANOVA with Kruskal–Wallis (non-parametric data) was then used to compare data among groups (control, G1, and G2). To compare data between the periodontal groups (G1 and G2), we used Student's unpaired *t*-test. Two-tailed *p*-values <0.05 were considered significant. The correlation between independent variables was calculated using Spearman's correlation coefficient (*r*), and linear regression was used to generate the line of best fit with 95% confidence intervals. The sensitivity and specificity of the relationship between two independent variables were determined using receiver operating characteristic (ROC) curve analysis.

## 3 Results

The results of the real-time PCR showed that the expression levels of all targeted molecules (ACE2, IL-6, miR-200c-3p, and miR-421-5p) were detected in the saliva of all participants with and without periodontitis.

### 3.1 Transcription levels and correlation analysis

**Figure 1:**
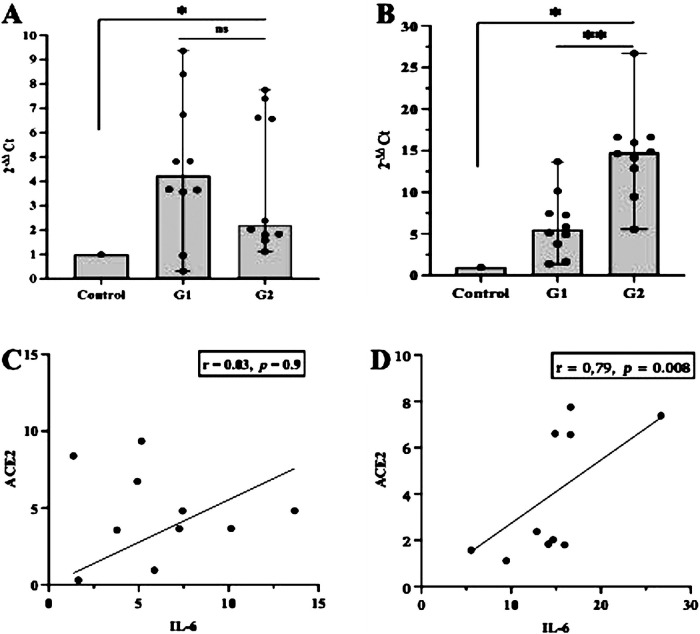
Evaluation of ACE2 and IL-6 expression and their correlation in different participant groups. The transcription levels of ACE2 mRNA and IL-6 were higher in the periodontitis groups (G1 or G2) than those in the control group. However, within the periodontitis groups, the different was only significant (*p *< 0.05) for the transcription levels of IL-6 **(A,B)**. In both periodontitis groups, the correlation between ACE2 and IL-6 was positive, but a strong association was only observed in participants with severe periodontitis (G2) **(C,D)**. *p < 0.05 between group; **p < 0.05 within group; ns, not significant. Spearman correlation coefficient (*r*) and exact *p*-values are depicted.

**Figure 2:**
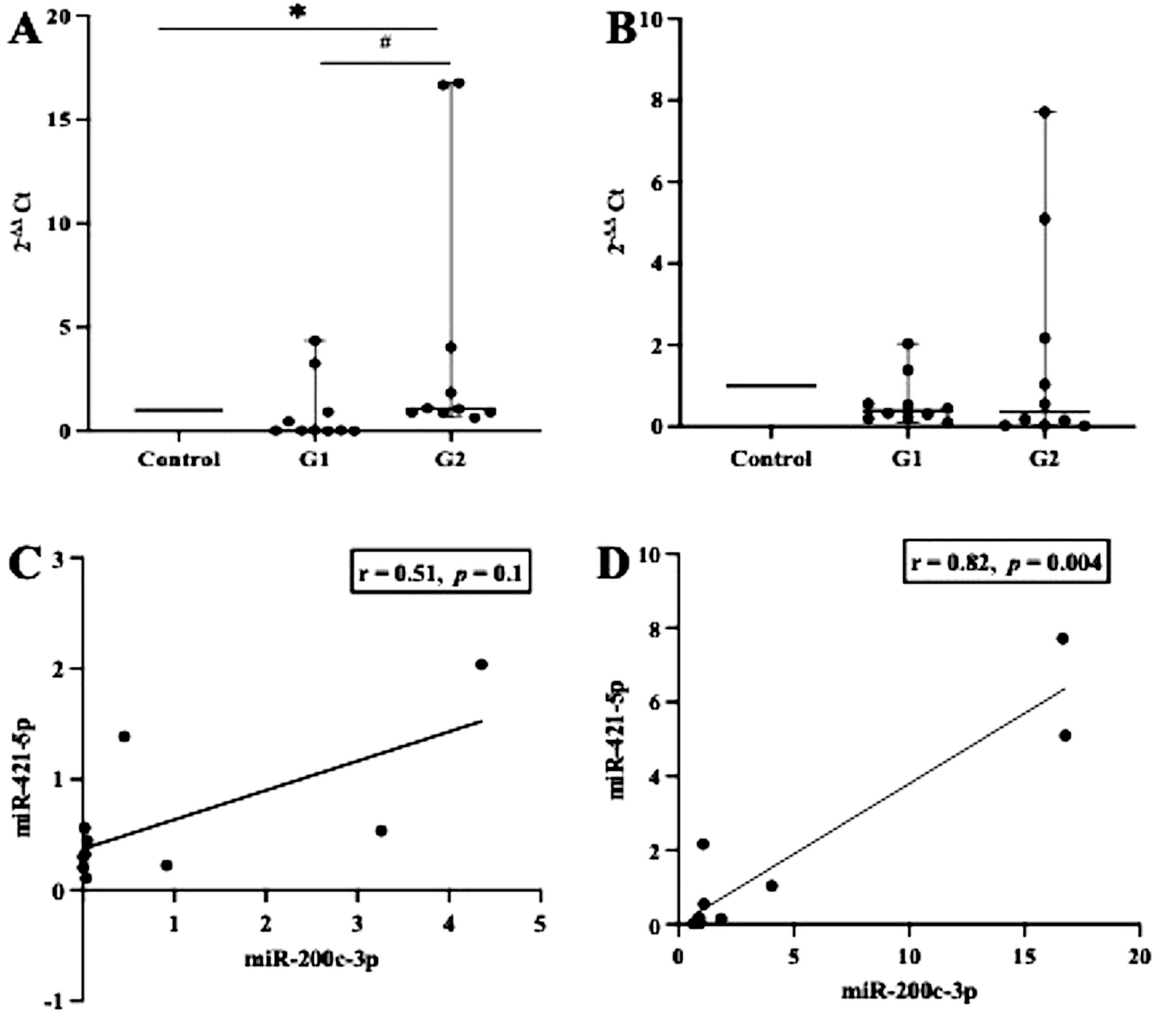
Transcription levels of miR-200c-3p and miR-421-5p and their correlation. In either between or within the groups tested (G1, G2, and control), miR-200c-3p transcription levels were significantly different, whereas miR-421-5p transcription levels were comparable **(A,B)**. These observations further indicate that the transcriptions of both miR-200c-3p and miR-421-5p show a positive linear association, but a strong correlation (*p *< 0.05) was only found in the G2 group **(C,D)**. *p < 0.05 between group; # not significant. Spearman correlation coefficient (*r*) and exact *p*-values are shown.

**Figure 3:**
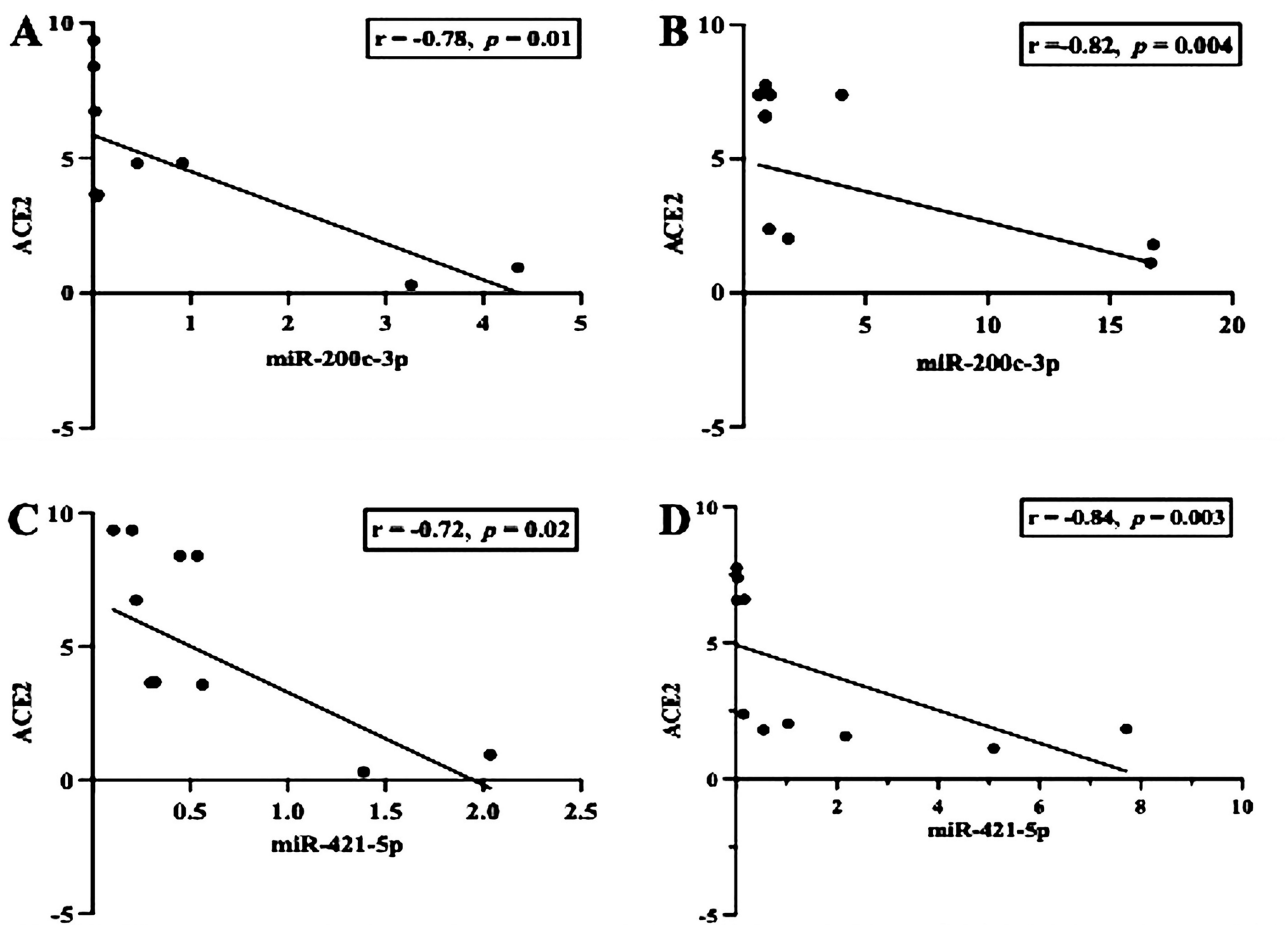
Scatter diagram illustrating the inverse correlation between the mRNA expression of ACE2 and miR-200c-3p/miR-421-5p. The observation indicates that, in either group observed (G1 and G2), the inverse correlation was strong **(A–D)**. Spearman correlation coefficient (*r*) and exact *p*-values are shown.

First, we examined whether inflammation activities in periodontal tissue upregulate the transcription of ACE2 and IL-6. As shown in Figures 1A,B, the level of ACE2 transcription was significantly upregulated in both groups studied (*p *< 0.05), but when we compared between the periodontitis groups (G1 and G2), the transcription was not significant (*p *> 0.05). In contrast, a significant increase in the transcription levels of IL-6 was identified in the G2 group compared with the participants in the G1 group (*p* < 0.0007). A Spearman correlation analysis was further performed to measure the strength and direction of association between ACE2 and IL-6. The results showed that in both periodontitis groups, there was a strong positive correlation between ACE2 and IL-6. The coefficient correlations in G1 and G2 were *r* = 0.03 (*p* = 0.9) and *r* = 0.79 (*p* = 0.008), respectively (Figures 1C,D). Given the regulatory role of miR-200c-3p and miR-421-5p in ACE2 expression (15), we further compared their transcription levels in the salivary exosome of both periodontitis groups and the controls. Again, the G2 group had a higher regulation of mir-200c-3p, which was statistically significant when compared with the G1 group (*p *< 0.01, Figure 2A). On the other hand, in both periodontitis groups, the transcription levels of miR-421-5p were comparable (*p *> 0.05, Figure 2B). However, in the G1 and G2 groups, the transcription levels of either miRNA were less than those observed in the control group. The Spearman test indicated that the correlation between both miRNAs was positive but not significant in the G1 group (*r* = 0.51, *p* = 0.1), but there was a strong positive correlation in the G2 group (*r* = 0.82, *p* = 0.004, Figures 2C,D). Subsequently, our data suggested that, irrespective of the periodontitis group, the inverse association between the ACE2 mRNA expression level and miR-200c-3p transcription level was significant. The coefficient correlations for G1 and G2 were *r* = −0.78 (*p* = 0.01) and *r* = −0.82 (*p *= 0.004), respectively (Figures 3A,B). Similarly, the SARS-CoV-2 receptor had a significant association with the miR-421-5p expression level, both in G1 (*r* = −0.72, *p* = 0.02) and G2 (*r* = −0.84, *p* = 0.003) (Figures 3C,D).

### 3.2 Evaluation of the area under the ROC curve

**Figure 4:**
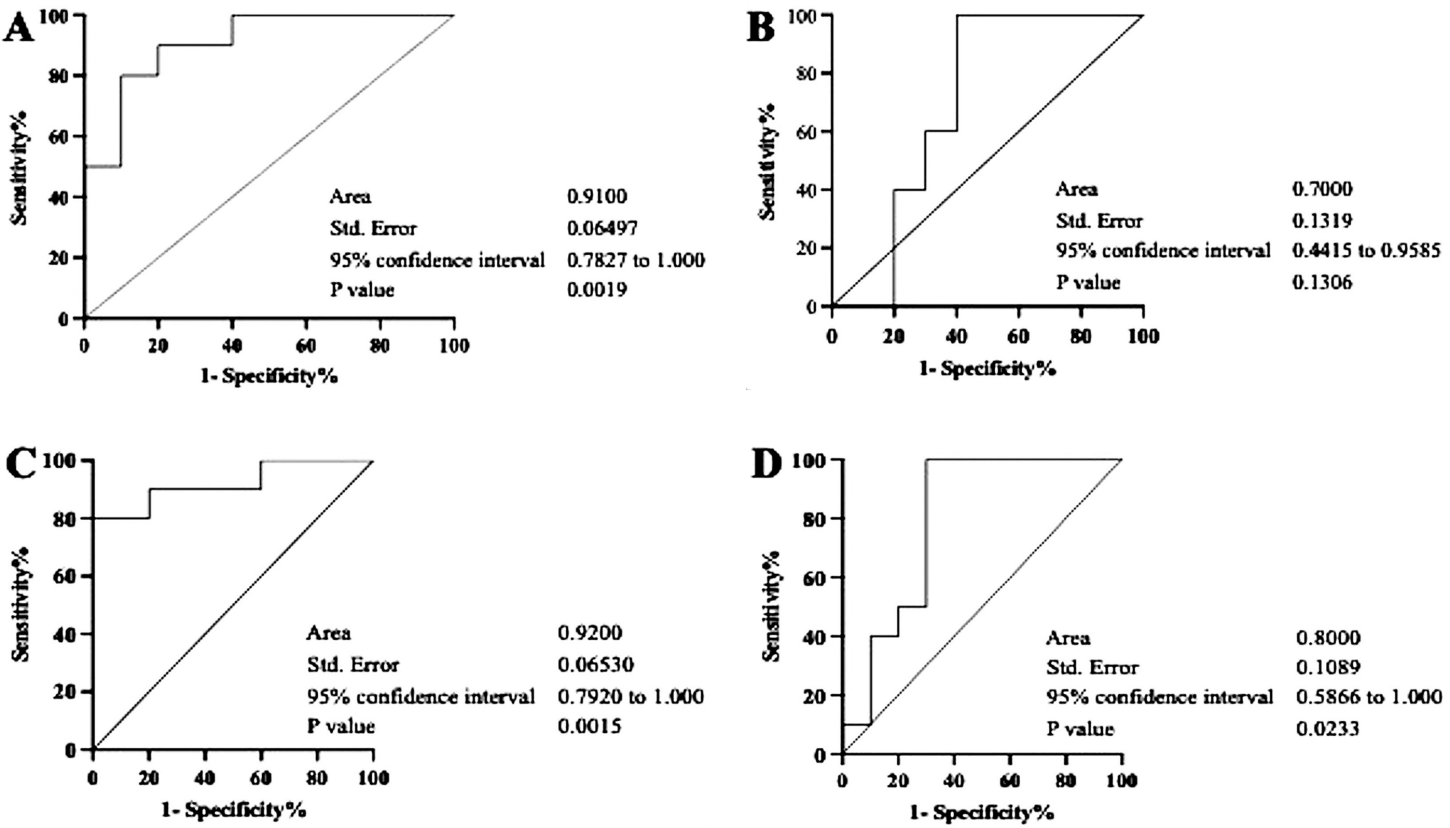
Diagnostic value analysis of the relationships between ACE2 and miR-200c-3p/miR-421-5p by ROC curves. The figures represent calculated ROC curves of the correlation between ACE2 and miR-200c-3p **(A,B)**, and between ACE2 and mir-421-5p **(C,D)**. Each of them exhibit characteristics of the sensitivity and specificity from the AUC values of ROC analysis. However, in both groups (G1 and G2), a consistently higher AUC value was only observed in the relationship between ACE2 and mir-421-5p.

By referring to the results, we further assessed the additional value to assess the prediction performance relationship of the interaction between the transcription levels of ACE2 and each of the miRNAs using ROC curve analysis (Figure 4). The area under the curve (AUC) of the relationships between ACE2 and miR-200c-3p was 0.91 with a *p*-value = 0.001 in G1 and 0.70 with a *p*-value = 0.1 in G2. For the interaction between ACE2 and miR-421-5p, the AUC was 0.92 with a *p*-value = 0.001 in G1 and 0.8 with a *p*-value = 0.02 in G2.

## 4 Discussion

ACE2 is a major contributing factor to SARS-CoV-2 infection (26), and it can be detected in saliva (8). In the current study, we found that soluble ACE2 can be detected in the saliva of vaccinated individuals with periodontitis at least 6 months post-vaccination, and the mRNA vaccine itself increases the endogenous synthesis of the S protein (27). It has been reported that after COVID-19 vaccination, the SARS-CoV-2 receptor (ACE2) can be membrane-bound or found as a soluble/circulating form within the body. In either form, it can still bind the receptor-binding domain of the S protein of SARS-CoV-2 (28). This protein is the most immunogenic antigen of the virus causing COVID-19 and is the major target for the immune system generated by the mRNA vaccine (29).

We assumed that periodontal inflammation occurred in our participants with periodontitis before the administration of COVID-19 mRNA vaccine, which raises the need to provide further evidence of the efficacy of the vaccine, more specifically in periodontal-disease-related individuals. Our data showed that the abundance of the soluble form of the SARS-CoV-2 receptor (sACE2) detected in saliva clearly differed among vaccinated participants with and without periodontitis. Hence, we questioned whether the relationships between the sACE2 and its modulator vary within the periodontitis groups. Indeed, we separated the periodontal status of our participants into moderate (G1) and severe (G2) periodontitis, with a non-periodontitis group included as a control. We noticed that, 6 months following vaccination, the booster dose elicited a salivary detectable sACE2, which was higher in periodontitis subjects than in controls. This result may suggest that the expression of *ACE2* mRNA in the oral environment appears to be accelerated by the presence of inflammation in periodontal tissue. However, the inflammation onset (moderate and severe periodontitis) may have no effect, as we observed that the ACE2 transcription levels were statistically comparable in both periodontitis groups (G1 and G2). Consistent with our data, a previous study reported that in plasma specimens, ACE2 activity remains elevated for more than 3 months after SARS-CoV-2 infection (30). Thus, it's feasible that the high concentration of sACE2 in our participants' saliva will prevent the S protein of SARS-CoV-2 from attaching to the membrane-bound ACE2. Considering that the membrane-bound ACE2 is the essential part of ACE2 for promoting SARS-CoV-2 infection (31), the presence of salivary sACE2 can be applicable as a surrogate marker of vaccine efficacy in vaccinated individuals with periodontitis.

In addition, the multifunctional cytokine (IL-6) was included as an independent variable in this study, as it plays an essential role in periodontal disease (32) and viral infection (33). Accordingly, previous research suggests that during immunization, miR-192 expression may change and have an impact on IL-6 levels (34). In this study, the median (range) of IL-6 in saliva was significantly higher in vaccinated participants with periodontitis than in controls and increased with the severity of disease. In addition, ACE2 transcription levels had a linear association with IL-6. Furthermore, it was demonstrated that the more severe periodontitis is, the greater the magnitude of the positive association between ACE2 and IL-6. This confirmed other reports that although COVID-19 vaccination does not affect plasma IL-6 levels (35), the salivary levels of sACE2 may reflect an inflammatory condition (28), which can be accelerated by periodontitis (36). Moreover, a previous report showed that elevated ACE2 expression occurred after treatment with lipopolysaccharide (LPS) from *Porphyromonas gingivalis* (37). This bacterium is an important periodontal pathogen that modulates IL-6 expression, although this is dependent on the bacterium's LPS isoform (38), and we did not include this in the evaluation in this study. Taken together, these results suggested that in the saliva of our participants, the two determinant factors (ACE2 and IL-6) associated with periodontitis in vaccinated individuals were relatively dependent. Thus, our finding from this study provided additional evidence that indicated that for at least 6 months post-COVID-19 vaccination, the expression of inflammation-associated ACE2/IL-6 increases when the phenotype of the periodontal tissue changes from no periodontitis to moderate periodontitis, and to severe periodontitis.

Considering the regulatory role of miRNA in the onset of inflammation and ACE2 activities (39), and owing to their crucial regulatory roles in the pathogenesis of periodontitis (40), we considered that the presence of sACE2 in the oral fluids of vaccinated participants with periodontitis provides additional information about the oral environment effect of the mRNA vaccine for COVID-19. Hence, it is tempting to speculate that the expression of the selected miRNAs (mir-200c-3-p and miRNA-421-5p) might be affected by the inflammation process in our participants with periodontitis, which we detected 6 months after they had received the COVID-19 mRNA vaccine. First, we found that there was a positive association between miRNA-200c-3p and miRNA 421-5p with a concurrent decrease in the two miRNAs when the periodontitis status altered from moderate to severe periodontitis in comparison with control participants. In addition, there was a significant difference in miR-200c-3p levels between the three groups 6 months post-COVID-19 vaccination. This miRNA was observed to decrease significantly in G1 compared with G2. This indicates its negative regulator for ACE2 (41) was associated with the severity of periodontal tissue inflammation. A similar trend was found in the expression of miR-421-5p. In both periodontitis groups (G1 and G2), the expression of miR-421-5p decreased compared with the control, although the difference was not significant (*p* = 0.1). Altogether, the obtained results may imply that with the onset of periodontitis in vaccinated individuals, a decrease in miR-200c-3p and miR-421-5p levels may prime the immune system, thereby influencing the response to the subsequent stimulus, which in our case was the COVID-19 mRNA vaccine. However, the underlying biopathological mechanism is as yet unclear.

On the other hand, the inverse correlation between miRNA-200c-3p/miRNA-421-5p and ACE2 may indicate that the low levels of either miRNA tested did not inhibit the expression of ACE2, which we observed in severe periodontitis has a strong association with the upregulation of IL-6. The current finding is in line with an earlier report describing the important role of the cytokine in periodontal destruction (32). Our finding further indicates that the role of miRNA-200c-3p/miRNA-421-5p as a negative regulator of ACE2 (42, 43) might be affected by inflammation and appeared to accelerate the expression of ACE2 in the periodontal microenvironment observed 6 months post-COVID-19 vaccination. Hence, to ascertain whether miRNA-200c-3p and miRNA-421-5p exhibit potential as predictors, the ROC curves were determined, and the AUC was considered. We found that only the association between ACE2 and miR-421-5p had a consistently statistically significant diagnostic strength, with AUC of 0.09 (*p* = 0.001) and 0.8 (*p* = 0.02) in moderate and severe periodontitis, respectively. This result suggests that salivary exosomal miR-421-5p has predictive power regarding the aggressiveness of periodontal inflammation and could be used as an independent prognostic biomarker for vaccine efficacy in periodontitis patients who received a COVID-19 mRNA vaccine, with a certain degree of accuracy. However, more studies are required to understand its role as a probable marker for the efficacy of COVID-19 mRNA vaccines.

## 5 Limitations of the study

Some intrinsic limitations should be noted in this study. First, as an observational study, only subjects aged <60 years were included. Age is the biological component that most closely correlates with the fluctuation in salivary miRNA levels (44). Also, in addition to the limited number of recruited participants, other potential confounders were not excluded. Gender, smoking habit, and coexisting conditions (e.g., diabetes) are examples of potential confounders. Smoking may upregulate ACE2 expression (45), and a reduction in miR-421 has been reported in diabetic patients (46). Therefore, the implication of some confounding variables may be miscalculated in predicting the result. In addition, periodontitis is a common oral infectious disease that is triggered by oral bacteria. It is important to analyze certain quantities of specific pathogens in the oral microbiota of patients that initiate inflammation, and we did not assess this in the current study.

## 6 Conclusions

Within the limitations of this study, our result revealed that the oral environment of vaccinated individuals with periodontitis maintains a distinctive relationship between ACE2 and its regulators (miR-200c-3p and miR-421-5p). This relationship could play a protective role against SARS-CoV2, and it is not interfered with by active and non-active periodontitis conditions for at least 6 months post-vaccination. Therefore, given the simplicity of saliva collection, detecting the soluble ACE2 in oral fluids may serve as an early tool for assessing population immunity and vaccine response, and it is particularly relevant as a potential non-invasive biomarker for predicting vaccine efficacy in vulnerable individuals (such as those with periodontitis) or when blood collection is not feasible. Moreover, ACE2 and its regulator might be relevant saliva markers for predicting the effectiveness of SARS-CoV-2 vaccines in other vulnerable populations, such as those with diabetes mellitus.

## Data Availability

The original contributions presented in the study are included in the article/Supplementary Material, further inquiries can be directed to the corresponding author.

## References

[1] Li Q Guan X Wu P Wang X Zhou L Tong Y Early transmission dynamics in Wuhan, China, of novel coronavirus-infected pneumonia. N Engl J Med. (2020) 382(13):1199–207. 10.1056/NEJMoa2001316 31995857 PMC7121484

[2] Burki T. Outbreak of coronavirus disease 2019. Lancet Infect Dis. (2020) 20(3):292–3. 10.1016/S1473-3099(20)30076-1 32078809 PMC7128260

[3] Song J Wu Y Yin X Zhang J. Relationship between periodontitis and COVID-19: a bidirectional two-sample Mendelian randomization study. Health Sci Rep. (2023) 6(8):e1413. 10.1002/hsr2.1413 37564397 PMC10409980

[4] Andrews M Gao H Datta S Katz J. Increased odds for COVID-19 infection among individuals with periodontal disease. Clin Oral Investig. (2023) 27(10):5925–33. 10.1007/s00784-023-05204-x 37606722

[5] Qi M Sun W Wang K Li W Lin J Gong J Periodontitis and COVID-19: immunological characteristics, related pathways, and association. Int J Mol Sci. (2023) 24(3). 10.3390/ijms24033012 PMC9917474 36769328

[6] Badran Z Gaudin A Struillou X Amador G Soueidan A. Periodontal pockets: a potential reservoir for SARS-CoV-2? Med Hypotheses. (2020) 143:109907. 10.1016/j.mehy.2020.109907 32504927 PMC7833827

[7] Gupta S Mohindra R Chauhan PK Singla V Goyal K Sahni V SARS-CoV-2 detection in gingival crevicular fluid. J Dent Res. (2021) 100(2):187–93. 10.1177/0022034520970536 33138663 PMC7642823

[8] Bachtiar EW Bachtiar BM Kusumaningrum A Sunarto H Soeroso Y Sulijaya B ACE2 expression in saliva of patients with COVID-19 and its association with *Candida albicans* and *Aggregatibacter actinomycetemcomitans*. F1000Res. (2022) 11:557. 10.12688/f1000research.111965.1 36112976 PMC9445561

[9] Bachtiar EW Soeroso Y Haerani N Ismah N Adiati EC Bachtiar BM. Immunoglobulin A response to SARS-CoV-2-N-protein potentially persists in oral fluids of patients with periodontitis six months after mRNA vaccine administration. J Dent Sci. (2024) 19(1):652–5. 10.1016/j.jds.2023.08.019 38303837 PMC10829667

[10] Bellavite P Ferraresi A Isidoro C. Immune response and molecular mechanisms of cardiovascular adverse effects of spike proteins from SARS-CoV-2 and mRNA vaccines. Biomedicines. (2023) 11(2). 10.3390/biomedicines11020451 PMC9953067 36830987

[11] Azzi L Dalla Gasperina D Veronesi G Shallak M Maurino V Baj A Mucosal immune response after the booster dose of the BNT162b2 COVID-19 vaccine. EBioMedicine. (2023) 88:104435. 10.1016/j.ebiom.2022.104435 36628844 PMC9828819

[12] Atherton LJ Jorquera PA Bakre AA Tripp RA. Determining immune and miRNA biomarkers related to respiratory syncytial virus (RSV) vaccine types. Front Immunol. (2019) 10:2323. 10.3389/fimmu.2019.02323 31649663 PMC6794384

[13] Mohammadi-Dehcheshmeh M Moghbeli SM Rahimirad S Alanazi IO Shehri ZSA Ebrahimie E. A transcription regulatory sequence in the 5′ untranslated region of SARSCoV-2 is vital for virus replication with an altered evolutionary pattern against human inhibitory MicroRNAs. Cells. (2021) 10(2). 10.3390/cells10020319 33557205 PMC7913991

[14] Kim SH Lee SY Lee YM Lee YK. MicroRNAs as biomarkers for dental diseases. Singapore Dent J. (2015) 36:18–22. 10.1016/j.sdj.2015.09.001 26684491

[15] Abdolahi S Hosseini M Rezaei R Mohebbi SR Rostami-Nejad M Mojarad EN Evaluation of miR-200c-3p and miR-421-5p levels during immune responses in the admitted and recovered COVID-19 subjects. Infect Genet Evol. (2022) 98:105207. 10.1016/j.meegid.2022.105207 34999004 PMC8730736

[16] Xu J Li Y Gan F Du Y Yao Y. Salivary glands: potential reservoirs for COVID-19 asymptomatic infection. J Dent Res. (2020) 99(8):989. 10.1177/0022034520918518 32271653

[17] Onabajo OO Banday AR Stanifer ML Yan W Obajemu A Santer DM Interferons and viruses induce a novel truncated ACE2 isoform and not the full-length SARS-CoV-2 receptor. Nat Genet. (2020) 52(12):1283–93. 10.1038/s41588-020-00731-9 33077916 PMC9377523

[18] Lambert DW Lambert LA Clarke NE Hooper NM Porter KE Turner AJ. Angiotensin-converting enzyme 2 is subject to post-transcriptional regulation by miR-421. Clin Sci (Lond). (2014) 127(4):243–9. 10.1042/CS20130420 24564768

[19] Blufstein A Behm C Gahn J Uitz O Naumovska I Moritz A Synergistic effects triggered by simultaneous toll-like receptor-2 and -3 activation in human periodontal ligament stem cells. J Periodontol. (2019) 90(10):1190–201. 10.1002/JPER.19-0005 31049957 PMC6852053

[20] Bachtiar BM Bachtiar EW Kusumaningrum A Sunarto H Soeroso Y Sulijaya B Porphyromonas gingivalis association with inflammatory markers and exosomal miRNA-155 in saliva of periodontitis patients with and without diabetes diagnosed with COVID-19. Saudi Dent J. (2023) 35(1):61–9. 10.1016/j.sdentj.2022.12.002 36540394 PMC9756571

[21] Bachtiar EW Soeroso Y Haerani N Ismah N Adiati EC Bachtiar BM. Immunoglobulin A response to SARS-CoV-2-N-protein potentially persists in oral fluids of patients with periodontitis six months after mRNA vaccine administration. J Dent Sci. (2024) 19(1):652–5. 10.1016/jjds202308019 38303837 PMC10829667

[22] Miller CS Foley JD Bailey AL Campell CL Humphries RL Christodoulides N Current developments in salivary diagnostics. Biomark Med. (2010) 4(1):171–89. 10.2217/bmm.09.68 20387312 PMC2857781

[23] Sakaguchi W Kubota N Shimizu T Saruta J Fuchida S Kawata A Existence of SARS-CoV-2 entry molecules in the oral cavity. Int J Mol Sci. (2020) 21(17). 10.3390/ijms21176000 PMC7503451 32825469

[24] Zhang MS Niu FW Li K. Proflavin suppresses the growth of human osteosarcoma MG63 cells through apoptosis and autophagy. Oncol Lett. (2015) 10(1):463–8. 10.3892/ol.2015.3206 26171052 PMC4487190

[25] Wu P Feng J Wang W. Expression of miR-155 and miR-146a in the saliva of patients with periodontitis and its clinical value. Am J Transl Res. (2021) 13(6):6670–7. 34306411 PMC8290752

[26] Hoffmann M Kleine-Weber H Schroeder S Kruger N Herrler T Erichsen S SARSCoV-2 cell entry depends on ACE2 and TMPRSS2 and is blocked by a clinically proven protease inhibitor. Cell. (2020) 181(2):271–80.e8. 10.1016/j.cell.2020.02.052 32142651 PMC7102627

[27] Angeli F Spanevello A Reboldi G Visca D Verdecchia P. SARS-CoV-2 vaccines: lights and shadows. Eur J Intern Med. (2021) 88:1–8. 10.1016/j.ejim.2021.04.019 33966930 PMC8084611

[28] Jia HP Look DC Tan P Shi L Hickey M Gakhar L Ectodomain shedding of angiotensin converting enzyme 2 in human airway epithelia. Am J Physiol Lung Cell Mol Physiol. (2009) 297(1):L84–96. 10.1152/ajplung.00071.2009 19411314 PMC2711803

[29] Chen Y Liu Q Guo D. Emerging coronaviruses: genome structure, replication, and pathogenesis. J Med Virol. (2020) 92(10):2249. 10.1002/jmv.26234 32881013 PMC7435528

[30] Patel SK Juno JA Lee WS Wragg KM Hogarth PM Kent SJ Plasma ACE2 activity is persistently elevated following SARS-CoV-2 infection: implications for COVID-19 pathogenesis and consequences. Eur Respir J. (2021) 57(5). 10.1183/13993003.03730-2020 PMC7830336 33479113

[31] Wan Y Shang J Graham R Baric RS Li F. Receptor recognition by the novel coronavirus from Wuhan: an analysis based on decade-long structural studies of SARS coronavirus. J Virol. (2020) 94(7). 10.1128/JVI.00127-20 PMC7081895 31996437

[32] Irwin CR Myrillas TT. The role of IL-6 in the pathogenesis of periodontal disease. Oral Dis. (1998) 4(1):43–7. 10.1111/j.1601-0825.1998.tb00255.x 9655045

[33] Soltani-Zangbar MS Parhizkar F Abdollahi M Shomali N Aghebati-Maleki L Shahmohammadi Farid S Immune system-related soluble mediators and COVID-19: basic mechanisms and clinical perspectives. Cell Commun Signal. (2022) 20(1):131. 10.1186/s12964-022-00948-7 36038915 PMC9421625

[34] Oshiumi H. Circulating extracellular vesicles carry immune regulatory miRNAs and regulate vaccine efficacy and local inflammatory response after vaccination. Front Immunol. (2021) 12:685344. 10.3389/fimmu.2021.685344 34211472 PMC8239358

[35] Langgartner D Winkler R Brunner-Weisser J Rohleder N Jarczok MN Gundel H COVID-19 vaccination exacerbates ex vivo IL-6 release from isolated PBMCs. Sci Rep. (2023) 13(1):9496. 10.1038/s41598-023-35731-2 37308487 PMC10261110

[36] Hajishengallis G Chavakis T. Local and systemic mechanisms linking periodontal disease and inflammatory comorbidities. Nat Rev Immunol. (2021) 21(7):426–40. 10.1038/s41577-020-00488-6 33510490 PMC7841384

[37] Sena K Furue K Setoguchi F Noguchi K. Altered expression of SARS-CoV-2 entry and processing genes by *Porphyromonas gingivalis*-derived lipopolysaccharide, inflammatory cytokines and prostaglandin E(2) in human gingival fibroblasts. Arch Oral Biol. (2021) 129:105201. 10.1016/j.archoralbio.2021.105201 34174588 PMC8215882

[38] Herath TD Wang Y Seneviratne CJ Lu Q Darveau RP Wang CY *Porphyromonas gingivalis* lipopolysaccharide lipid A heterogeneity differentially modulates the expression of IL-6 and IL-8 in human gingival fibroblasts. J Clin Periodontol. (2011) 38(8):694–701. 10.1111/j.1600-051X.2011.01741.x 21752043

[39] Huang N Perez P Kato T Mikami Y Okuda K Gilmore RC SARS-CoV-2 infection of the oral cavity and saliva. Nat Med. (2021) 27(5):892–903. 10.1038/s41591-021-01296-8 33767405 PMC8240394

[40] Santonocito S Polizzi A Palazzo G Isola G. The emerging role of microRNA in periodontitis: pathophysiology, clinical potential and future molecular perspectives. Int J Mol Sci. (2021) 22(11). 10.3390/ijms22115456 PMC8196859 34064286

[41] Gupta R Radicioni G Abdelwahab S Dang H Carpenter J Chua M Intercellular communication between airway epithelial cells is mediated by exosome-like vesicles. Am J Respir Cell Mol Biol. (2019) 60(2):209–20. 10.1165/rcmb.2018-0156OC 30230353 PMC6376407

[42] Liu Q Du J Yu X Xu J Huang F Li X miRNA-200c-3p is crucial in acute respiratory distress syndrome. Cell Discov. (2017) 3:17021. 10.1038/celldisc.2017.21 28690868 PMC5485385

[43] Trojanowicz B Imdahl T Ulrich C Fiedler R Girndt M. Circulating miR-421 targeting leucocytic angiotensin converting enzyme 2 is elevated in patients with chronic kidney disease. Nephron. (2019) 141(1):61–74. 10.1159/000493805 30326474

[44] Sullivan R Montgomery A Scipioni A Jhaveri P Schmidt AT Hicks SD. Confounding factors impacting microRNA expression in human saliva: methodological and biological considerations. Genes (Basel). (2022) 13(10). 10.3390/genes13101874 36292760 PMC9602126

[45] Smith JC Sausville EL Girish V Yuan ML Vasudevan A John KM Cigarette smoke exposure and inflammatory signaling increase the expression of the SARS-CoV-2 receptor ACE2 in the respiratory tract. Dev Cell. (2020) 53(5):514–29.e3. 10.1016/j.devcel.2020.05.012 32425701 PMC7229915

[46] Elemam NM Hasswan H Aljaibeji H Sulaiman N. Circulating soluble ACE2 and upstream microRNA expressions in serum of type 2 diabetes mellitus patients. Int J Mol Sci. (2021) 22(10). 10.3390/ijms22105263 34067683 PMC8156444

